# Case report-immune recovery posterior scleritis in a HIV positive patient

**DOI:** 10.1186/s12886-020-01529-3

**Published:** 2020-07-02

**Authors:** Xin Che, Jing Jiang, Yiwen Qian, Qingjian Li, Yu Zhang, Zhiliang Wang

**Affiliations:** Department of Ophthalmology, Huashan Hospital of Fudan University, No. 12, Middle Urumqi Road, Shanghai, 200040 China

**Keywords:** HIV, Immune recovery posterior scleritis, HAART, Treatment, Case report

## Abstract

**Background:**

Posterior scleritis is an uncommon vision-threatening disorder that is often recurrent and difficult to cure due to its complex etiology. In HIV patients, posterior scleritis may develop several months after significant rise in CD4+ T-lymphocyte, even after several years, which may be diagnosed as late-onset immune recovery scleritis.

**Case presentation:**

Here we report a case of posterior scleritis in a HIV positive patient who presented with severe periocular pain and decreased vision in the left eye, with exudative retinal detachment and optic nerve involvement.

**Conclusions:**

Early differential diagnosis of immune recovery posterior scleritis and intensive corticosteroids treatment, can prevent vision loss effectively in HIV patients.

## Background

Posterior scleritis refers to a heterogeneous group of disorders characterized by chronic inflammatory response, which is associated with exudative retinal detachment and optic nerve involvement. It is commonly confounded with ocular tumors and other inflammatory ocular disorders, like choroidal tumor and Vogt-Koyanagi-Harada syndrome [1]. A part of scleritis is due to infections [2–4], about 10% scleritis is associated with systemic disorders like rheumatic diseases and most of the origin is idiopathic [5, 6].

Immune recovery scleritis, is a noninfectious scleral inflammation which develops in patients with CMV retinitis or other intraocular infections after substantial increase in CD4+ T-lymphocyte. Immune recovery scleritis in human immunodeficiency virus (HIV) patients is uncommon, which may develop several months after the highly active anti-retroviral therapy (HAART) initiation when the immune function is recovered [7].

Early diagnosis and intervention in treating immune recovery scleritis is necessary to avoid vision loss. Therapeutic strategies focus on sub-conjunctival triamcinolone injections for anterior scleritis, and systemic corticosteroids with or without systemic immunosuppressive drugs for severe scleritis [8]. But so far, treatment strategies for HIV patients with posterior scleritis remain controversial.

In this case, we report a HIV patient who was diagnosed with presumed immune recovery posterior scleritis unassociated with infectious etiology or rheumatologic diseases.

## Case presentation

**Fig. 1:**
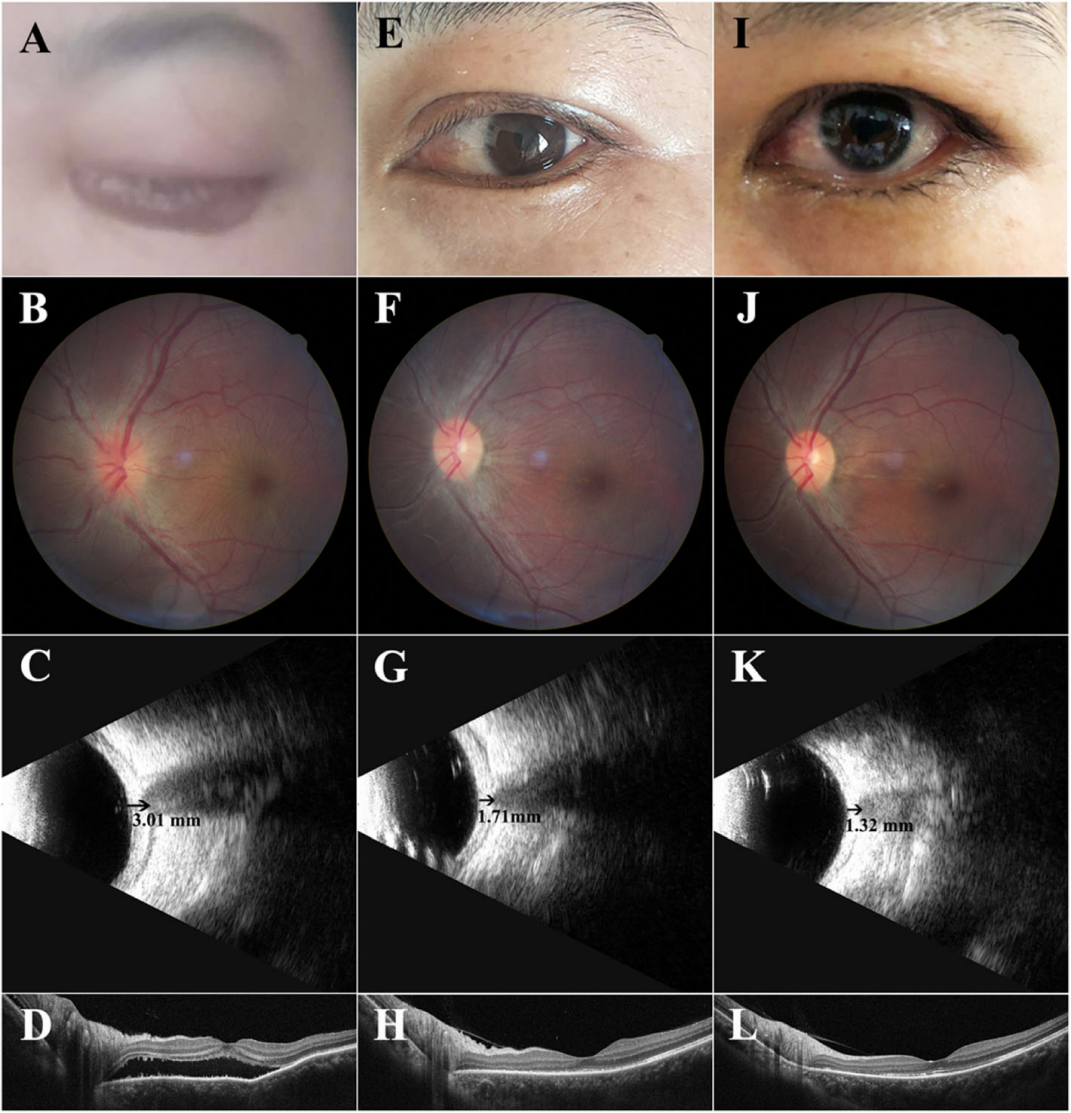
Anterior segment photography of the left eye. **a.** Severe chemosis, subconjunctival hemorrhage at the first visit. **e.** Regression of chemosis and complete resolution of conjunctival redness 1 month later. **i.** No obvious abnormality 6 month later. Fundus photograph of the left eye. **b.** Presence of optic nerve swelling and chorioretinal folds at the first visit. **f.** Partial regression of optic nerve swelling and progressive resolution of chorioretinal folds 1 month later. **j.** Absolute regression of optic nerve swelling and chorioretinal folds 6 month later. B-ultrasound of the left eye. **c.** Retrobulbar edema with the typical “T” sign at the first visit. **g.** Reduction of retrobulbar edema 1 month later. **k.** Mild retrobulbar edema 6 month later. OCT of the left eye. **d.** Exudative retinal detachment at the first visit. **h.** Regression of retinal detachment 1 month later. **l.** Normal retinal structure 6 month later.

A 37-year-old woman came to our eye clinic with complaints history of ocular and periocular pain in the left eye, irradiating to the ipsilateral forehead, and decreased vision with a central scotoma. Her best-corrected visual acuity (BCVA) was 20/20 (OD) and 20/67 (OS), with intraocular pressure (IOP) of 11 mmHg (OD) and 8 mmHg (OS). Examination of the right eye was normal. Anterior segment examination of the left eye revealed severe chemosis, subconjunctival hemorrhage (Fig. 1a). Fundus examination showed the disc edema and retinochoroidal folds (Fig.1b). B-ultrasound (Fig.1c) and optical coherence tomography (OCT) (Fig. 1d) revealed retrobulbar edema with typical “T” sign and exudative retinal detachment, suggesting posterior scleritis. She was systemically asymptomatic, with a normal hematology index, negative rheumatoid factors, complement fractions C3 and C4, ANA, ANCA, circulating immunocomplexes and a normal orbital magnetic resonance imaging (MRI). Serologic tests for syphilis, tuberculosis, hepatitis B and C were negative.

She was under consistent HAART for 3 years, during which her CD4 counts significantly peaked from 103 cells/µL to 432 cells/µL. When she presented to our hospital with severe posterior scleritis, the CD4 count has further elevated to 698 cells/µL (normal range 400–1300 cells/µL) and the HIV viral load was tested below the detection level.

The patient was subconjunctivally injected with Triamcinolone (1 mg) at our outpatient service immediately, but showed no signs of improvement. One week later, she underwent a diagnostic pars plana vitrectomy to rule out infectious etiology, with an intravitreal injection of 4 mg Triamcinolone. Molecular analysis of vitreous aspirate detected low titer of Aspergillus sydowii DNA, but was neglected as the patient did not show symptoms of endophthalmitis, which was presumed as a non-viable infection. After vitrectomy, topical 0.1% Dexamethasone eyedrops four times daily and ointment every night was prescribed. Surprisingly, within 5 days her vision improved.

During her 1 month follow-up, the left eye showed a visual acuity of 20/20, chemosis and subconjunctival hemorrhage was completely resolved (Fig.1e), the optic disc was normal while retinochoroidal folds still existed (Fig.1f). Ultrasound (Fig.1g) and OCT (Fig. 1h) showed a reduction of retrobulbar edema and complete regression of retinal detachment. Since the patient showed an excellent therapeutic response, the topical 0.1% Dexamethasone eyedrops and ointment was continued for 6 months. Then the follow-up BCVA was 20/20, the retrobulbar inflammation subsided (Fig.1i, j, l), B-ultrasound showed mild edema (Fig. 1k).

## Discussion and conclusions

Immune reconstitution inflammatory syndrome (IRIS) is commonly observed in HAART initiated HIV patients wherein the preexisting infection and inflammation worsens due to immune recovery. A variety of opportunistic pathogens including viruses, fungi and bacteria are associated with the development of IRIS [9]. Among various IRIS, immune recovery uveitis (IRU) is the common condition observed in HIV patients, while posterior scleritis rarely occur in HIV patients [10]. Literature survey showed only one case report on immune recovery scleritis in HIV patient [11].

To the best of our knowledge, this is the second case report presenting immune recovery scleritis in HIV patient. Although the vitreous biopsy was tested Aspergillus positive in our case, the infectious etiology was ruled out as symptoms of vasculitis and vitritis were absent and the patient responded well to topical corticosteroid treatment. Immune recovery was observed to be the only risk factor associated with posterior scleritis as the CD4 cells increased after HAART. However, for this patient, idiopathic scleritis could not be excluded.

IRIS is divided into early-onset (< 3 months from HAART initiation) and late-onset presentation (> 3 months after HAART initiation). Early-onset IRIS is best explained by an increased immune response to either subclinical or partially treated opportunistic infections, late-onset is characterized by an immune reaction induced against non-viable pathogens, which has been reported even years after onset of HAART administration [12, 13]. Our case is a presumed example of late-onset immune recovery posterior scleritis which has developed after 3 years of HAART.

During the early stage of infection, the vitreous inflammation may not be obvious. Considering our case is a HIV patient, diagnostic vitrectomy is important to do the PCR analysis to rule out infectious origin. Meanwhile, for the posterior scleritis, vitrectomy could eliminate inflammatory mediators in the eye, avoid cystoid macular edema, and could reduce the need for systemic long-term steroid, especially for the patients whose general situation may exacerbate after receiving systemic corticosteroids.

With the development of HAART globally, clinicians may encounter increasing numbers of individuals with IRIS. The ophthalmologist should be alert in considering IRIS as potential risk factor of posterior scleritis even several years after the initiation of HAART.

## Data Availability

Not applicable.

## Abbreviations

HIV: Human immunodeficiency virus
HAART: Highly active anti-retroviral therapy
BCVA: Best-corrected visual acuity
OCT: Optical coherence tomography
MRI: Magnetic resonance imaging
IRIS: Immune reconstitution inflammatory syndrome
IRU: Immune recovery uveitis
